# SHP2-Mediated Signal Networks in Stem Cell Homeostasis and Dysfunction

**DOI:** 10.1155/2018/8351374

**Published:** 2018-06-10

**Authors:** Chen Kan, Fan Yang, Siying Wang

**Affiliations:** School of Basic Medical Sciences, Anhui Medical University, 81 Meishan Road, Hefei 230022, China

## Abstract

Stem cells, including embryonic stem cells (ESCs) and adult stem cells, play a central role in mammal organism development and homeostasis. They have two unique properties: the capacity for self-renewal and the ability to differentiate into many specialized cell types. Src homology region 2- (SH2-) containing protein tyrosine phosphatase 2 (SHP-2), a nonreceptor protein tyrosine phosphatase encoded by protein tyrosine phosphatase nonreceptor type 11 gene (PTPN11), regulates multicellular differentiation, proliferation, and survival through numerous conserved signal pathways. Gain-of-function (GOF) or loss-of-function (LOF) SHP2 in various cells, especially for stem cells, disrupt organism self-balance and lead to a plethora of diseases, such as cancer, maldevelopment, and excessive hyperblastosis. However, the exact mechanisms of SHP2 dysfunction in stem cells remain unclear. In this review, we intended to raise the attention and clarify the framework of SHP2-mediated signal pathways in various stem cells. Establishment of integrated signal architecture, from ESCs to adult stem cells, will help us to understand the changes of dynamic, multilayered pathways in response to SHP2 dysfunction. Overall, better understanding the functions of SHP2 in stem cells provides a new avenue to treat SHP2-associated diseases.

## 1. Introduction

Stem cells, derived from embryo and adult tissues, can be differentiated into various terminal-differentiated cells involved in the multiple processes, including development and homeostasis. Evans and Kaufman first identified and isolated mouse ESCs in 1981 [1, 2]. Later, Thomson et al. isolated and cultured human ESCs in 1998 [3]. The establishment of ESCs has generated a great deal of excitement in the field of regenerative medicine, and the name was coined by Kaiser in 1992 [4]. Surprisingly, Yamanaka's group announced that adult cells, transfected by four specific genes, could be reversed into pluripotent stem cells in 2006 [5, 6]. This work completely revolutionized the original idea about the stem cells. On the other hand, identification of adult stem cells resided in adult tissue, without concerns of teratomas, ethical issue, and other challenges, has moved the stem cells field forward. Hematopoietic stem cells (HSCs) are the stem cells that give rise to all blood cells were first discovered in the 1950s [7]. Malignant proliferation of hematopoietic stem/progenitor cells, caused by genetic or environmental factors, leads to leukemia. Another extensively studied adult stem cells, mesenchymal stem cells (MSCs), discovered by Friedenstein and colleagues [8], are thought to have tremendous potential in tissue engineering owing to their capacity of multilineage differentiation.

Stem cells give rise to various functional cells and maintain organism homeostasis. Unsurprisingly, disturbance of stem cell function usually causes severe disorders, such as leukemia, malignantly solid tumors, and other degenerative diseases. Several conserved signaling pathways, depended on cell types and development stages, are responsible for the versatile capacity of many stem cells. For example, concerted leukemia inhibitory factor (LIF)/bone morphogenetic protein (BMP) signaling maintains mouse ESCs differentiation and self-renewal [9]. Later, Wnt and BMP signals are demonstrated to contribute to HSCs expansion and self-renewal [10], while BMP/hedgehog signaling mediates the osteogenic or chondrogenic differentiation of MSCs, respectively [11, 12]. In the context of cancers, Wnt and hedgehog signaling pathways often regulate cancer stem cells (CSCs) self-renewal, whereas BMP signaling is closely associated with CSCs differentiation [9]. Although many signaling cascades which are the chain of biochemical events along a signaling pathway were investigated in stem cells, there are largely unknown parts in stem cells signaling network.

In this review, we focus on the functional role of SHP2 in stem cells. SHP2 is a ubiquitous multidomain nonreceptor protein tyrosine phosphatase (PTP). This PTP contains two tandem Src homology-2 (SH2) domains, which function as phosphotyrosine-binding domains and mediate the interaction of this PTP with its substrates and play a central role in multiple signaling pathways. Dysregulation of SHP2 causes cancers, diabetes, and Noonan syndrome (NS) [13], which widely affected the human organ systems such as the heart, lung, blood, skeleton, brain, and gastrointestinal system. More recently, Dong et al. reported that MSCs with GOF mutations of SHP2 (PTPN11) regulated HSCs or myeloid progenitors malignant proliferation in bone marrow microenvironment and eventually promoted myeloproliferative neoplasm (MPN) [14]. Additionally, others also proved that SHP2 contributed to solid tumor progression through facilitating cancer stem/initiating cells expansion [15–17]. These findings suggest that understanding SHP2 signaling in stem cells is important to further explore its underlying role in causing disease.

Therefore, we updated the fundamental mechanisms of SHP2 in stem cells including ESCs, HSCs, MSCs, neural stem cells (NSCs), cancer stem cells (CSCs), and other tissue-specific stem cells. Furthermore, we integrated the whole underlying signaling pathways into a network from ESCs to adult stem cells. Our discussion will highlight the importance of stem cells-associated SHP2 as well as provide a novel insight for translational research and clinical therapies.

### 1.1. SHP2 in Embryonic Stem Cells

SHP2, distinguished from several conserved transcription factors for maintaining ESCs stemness, is well known to play an important role as the molecular switch of ESCs self-renewal and differentiation [18]. Using genetic lineage tracing, Saxton and Pawson found that in SHP2-deficient mouse, ESCs were accumulated in the posterior epiblast of the gastrulating embryo rather than migrating through primitive streak and showed limited differentiation capacity toward ectoderm tissue. In addition, they found that loss-of-function (LOF) SHP2 ends up failing to respond to fibroblast growth factor (FGF), an essential chemotactic cytokine in development, and eventually led to aberrant phenotypes [19]. Furthermore, in chimaeric mouse embryos with double mutations of SHP2 and FGF receptors, mutant cells failed to participate in the formation of the limb, suggesting SHP2-mediated FGF signaling pathway might mediate this effect [20].

Evidence so far suggests that SHP2 seems to be a negative regulator of ESCs self-renewal. For example, in undifferentiated mouse ESCs, SHP2 functions as a downstream regulator of cytokine receptors, receptor tyrosine kinases (RTKs), and their coreceptors, such as leukemia inhibitory factor (LIF) receptor and zeta-chain-associated protein kinase (Zap70). Zap70 activates SHP2 and phosphorylates extracellular signal-regulated kinase (ERK) signaling. Upregulation of ERK represses leukemia inhibitory factor (LIF) receptor expression and inhibits the janus kinase 1 (JAK1)/signal transducer and activator of transcription 3 (STAT3) pathway which is essential in ESCs self-renewal. Inhibition of SHP2 facilitates maintenance of mouse ESCs stemness due to increased STAT3 activity [21]. In contrast, Matsuda et al. showed that LIF-associated mouse ESCs self-renewal was responsible for STAT3 activation but not gp130- (a subunit of LIF receptor) mediated SHP2 signals [22]. This critical result indicates that SHP2/STAT3 pathway plays a crucial role in ESCs self-renewal. However, when SHP2 is impaired, other compensated signals, for example, ERK, may be implicated to regulate STAT3 expression [23].

**Figure 1:**
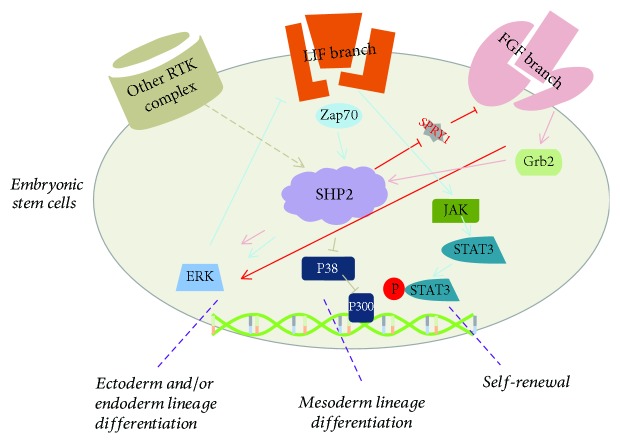
SHP2 is a molecular switch of ESC self-renewal and/or differentiation. LIF-JAK-STAT3 signaling manipulates the ESC self-renewal, upregulation of SHP2 facilitates the ERK signals and inhibits LIF-JAK-STAT3 signal, while inhibition of SHP2 maintains the capacity of ESC self-renewal. Upon ESC differentiation, SHP2 acts a downstream effector of RTKs and triggers distinct major signal cascades and eventually regulates target gene expression. For example, SHP2 activates ERK and promotes ESC differentiation toward ectoderm and/or endoderm lineage differentiation.

Consistently, other investigators have demonstrated that SHP2 also plays an important role in mouse ESCs differentiation. For example, during neural (ectoderm) differentiation, SHP2 suppresses the activity of sprouty1 which is an antagonist of FGF pathway. Inactivation of sprouty1 enhances the FGF/ERK signaling and eventually promotes ESCs differentiation into neural cells [24]. In addition, FGF4 directs growth factor receptor-bound protein2 (Grb2) to bind SHP2 and further activates ERK pathway which is sufficient for differentiation for the essential primitive endoderm lineage from ESCs [25]. For mesoderm differentiation, SHP2 also exhibits critical function. He et al. found that SHP2 suppressed P38 mitogen-activated protein kinases (P38 MAPK) activation, leading to stabilization of p300 which is an important transcriptional coactivator and enhanced ESCs differentiation toward adipocytes via targeting peroxisome proliferator-activated receptors-*γ* (PPAR*γ*) [26]. SHP2 also regulates VE-cadherin expression through sprouty1 pathway but not FGF/ERK pathway in ESCs differentiation toward endothelial cells [24]. Together, these results indicate that SHP2 mediates distinct signal cascades in a context-dependent manner, which is extremely important in ESCs homeostasis (Figure 1).

### 1.2. SHP2 in Hematopoietic Stem Cells

SHP2, initially reported by Qu et al. in hematopoietic cells [27, 28], is essential for regulating HSCs capacities, that is, SHP2 is implicated to regulate HSCs stemness, homing, and survival [29–31]. On the other hand, myriad hematopoietic diseases, for example, juvenile myelomonocytic leukemia (JMML), are closely associated with dysfunction of SHP2 in HSCs. However, the molecular mechanisms underlying HSCs function are still largely unknown.

**Figure 2:**
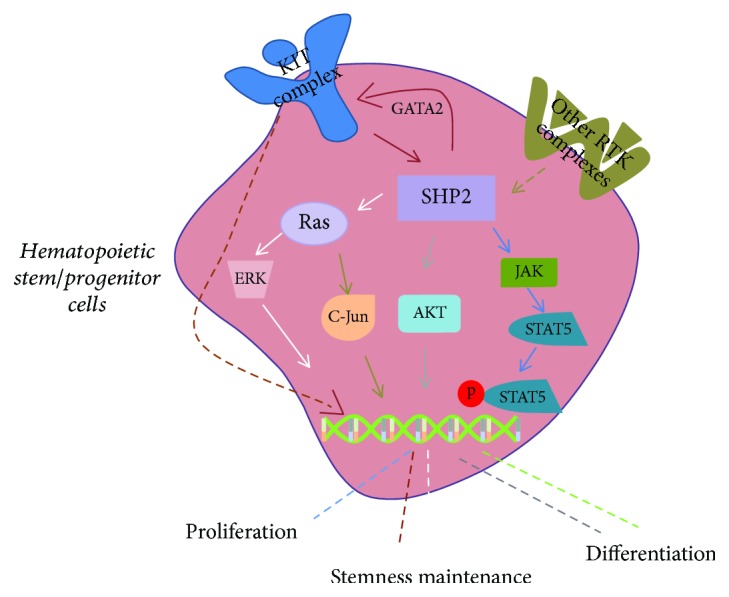
SHP2 plays a key role in HSC differentiation and self-renewal. Kit, an important surface marker of hematopoietic cells, plays a pivotal role in HSC self-renewal. Kit-SHP2-GATA2 loop positively regulates HSC stemness. In addition, ERK may be functioned as a downstream effector of SHP2 in HSC self-renewal. During HSC differentiation, SHP2/AKT may be involved in the myeloid differentiation and SHP2/GATA1 signaling regulates HSC differentiation into erythroid cells. Besides, SHP2/JAK/STAT5 signaling is responsible for HSC proliferation.

Generally, HSCs in red bone marrow are quiescent and a cohort of signals regulates HSCs functions, including differentiation, self-renewal, homing, and survival. SHP2 has been demonstrated to play a key role in HSCs homeostasis. For example, deficiency of SHP2 causes defective ERK in HSCs and leads to cytopenia in mouse [29]. Mechanistically, receptor tyrosine kinases (RTKs) could act as an upstream regulator of SHP2, that is, activate SHP2 and subsequently downstream signal cascade to regulate the target gene expression. Of note, upon different RTK activations, SHP2 exhibits distinct functions (Figure 2). For example, Kit, also known as mast/stem cell growth factor receptor, activates SHP2 and phosphorylates Gata2 and eventually increases Kit expression. This positive feedback loop promotes HSC homing, survival, and self-renewal [29]. In fact, distinct signaling cascades mediated by SHP2 show diverse functions in HSCs, that is, SHP2/Ras/ERK pathway is critical for HSCs maintenance [31], and SHP2/AKT or SHP2/JAK/STAT5 signaling is responsible for HSCs differentiation and survival, respectively [30]. Interestingly, SHP2 has limited effects on differentiation of myeloid progenitors. Under macrophage colony-stimulating factor (M-CSF) stimulation, myeloid progenitors prefer to monopoiesis via activating ERK and thereby induce higher levels of c-Fos and phospho-C/EBPa (S21). In contrast, granulocyte colony-stimulating factor (G-CSF) stimulates myeloid progenitors to differentiate into granulocytes through activating SHP2/STAT3 signaling [32]. In addition, Gata1, an essential element of erythroid differentiation of HSCs, epigenetically suppresses SHP2 function and eventually promotes erythropoiesis [33]. Overall, LOF SHP2 results in reduced quiescence, increased apoptosis, and substantially reduced HSCs-repopulating capacity.

GOF mutations of SHP2 (SHP2^D61Y^ and SHP2^E76K^), on the other hand, cause erythrocyte-associated diseases [34, 35] and myeloproliferative neoplasm. There are at least three mechanisms that have been reported: (1) Activating SHP2 causes hematopoietic progenitor aberrant differentiation and proliferation. Qu et al. first established induced SHP2^E76K^ knock-in (global knock-in causes embryonic lethality) mouse model and found tissue-specific knock-in ptpn11^E76K/+^ mutation in lineage-committed myeloid and T lymphoid and B lymphoid progenitors results in AML, T-ALL, and B-ALL, respectively. Furthermore, they indicated that hyperactivation of SHP2 was distributed into centrosomes and caused centrosome amplification and genomic instability which lead to malignant proliferation of HSCs or myeloid progenitors [36]. Consistently, SHP2^D61Y^ mutation in myeloid progenitors and granulocyte-monocyte progenitors also leads to autonomous HSCs proliferation. Additionally, upon stimulation of stem cell factor (SCF), ERK and AKT signaling is activated, while STAT5 signal is responsible for granulocyte-macrophage colony-stimulating factor (GM-CSF)-evoked aberrant cell proliferation [34, 37, 38]. During HSCs differentiation, GOF SHP2, including SHP2^E76K^ and SHP2^D61Y^, induces Ras hyperactivation and promotes c-Jun phosphorylation and expression and eventually leads to aberrant monocytic differentiation in hematopoietic progenitors [39]. Interestingly, SHP2 activation leads to inhibition of interferon consensus sequence-binding protein (Icsbp), which is an interferon regulatory transcription factor, and subsequently increases growth arrest specific 2 (Gas2), a calpain inhibitor, and finally elevates STAT5 expression. These findings suggest that signaling loop (SHP2/Icsbp/Gas2/calpain/STAT5) was essential for myeloid leukemia [40]. (2) Activating SHP2 promotes hematopoietic cells survival. HSCs with LOF or GOF SHP2 show differential effects on HSCs survival, that is, LOF SHP2 promotes HSCs apoptosis and GOF SHP2 controls the survival and maintenance of HSCs. For example, SHP2^E76K^ mutation increases leukemia stem cells proportion and upregulates antiapoptotic gene expression [41]. Yang et al. demonstrated that hematopoietic progenitors bearing the GOF SHP2 mutants resided in the S or G2 phase of the cell cycle. Biochemical analysis also showed that expressions of antiapoptotic genes, such as cyclinD1, Bcl2, and BclXl, were increased [42]. (3) Activating mutations of SHP2 in myeloid progenitors enhance reactive oxygen species (ROS) production and contribute to myeloproliferative disorder. ROS, a natural byproduct of metabolism of oxygen, is demonstrated to be closely associated with NS. Oxidative phosphorylation complexes are responsible for SHP2 function in NS patients [43]. Further studies indicate that mitochondrial protein P135, functioned as a substrate of SHP2, promotes myeloid progenitors malignant proliferation [44].

### 1.3. SHP2 in Mesenchymal Stem Cells

**Figure 3:**
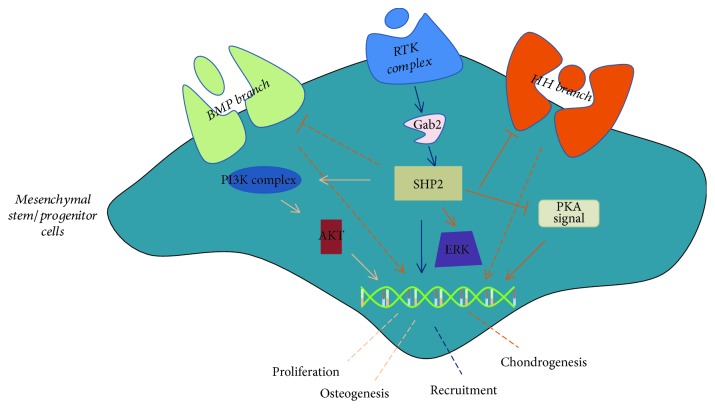
SHP2 regulates MSC differentiation, proliferation, and recruitment. SHP2 likely promotes MSC proliferation and/or osteogenic differentiation through PI3K-AKT signal pathway. In addition, SHP2 negatively regulates MSC chondrogenesis by enhancing IHH, BMP, and PKA signaling and eventually upregulates SOX9 and chondrogenesis-associated gene expression. Also, GOF SHP2 activates CCL3 signaling in MSCs and aberrantly recruits HSCs for MPN.

MSCs, known as the supportive cells of HSCs, are usually associated with distinct diseases, such as heterotopic ossification (HO) [11, 12] or osteoporosis [45]. As mentioned above, SHP2 plays an indispensable role in mesoderm development and more recent studies have shown that SHP2 regulates MSCs differentiation, proliferation, and recruitment (Figure 3). For example, application of chlorogenic acid (CGA) promotes MSCs osteogenic differentiation through SHP2/AKT signaling [46]. Moreover, inhibition of SHP2 rarely affects MSCs adipogenic differentiation, but inhibits MSCs proliferation through AKT/cycling D1 pathways [47]. Fan et al. also demonstrated that SHP2 promotes MSCs osteogenic differentiation both in vitro and in vivo [48]. Surprisingly, Yang et al. identified a novel marker of mesenchymal progenitors, namely, chondroid cells and the cathepsin K (Ctsk)-cre-labeled cells, and indicated that SHP2-deficient Ctsk-expressing cells could form chondroid neoplasms. Furthermore, they demonstrated that LOF SHP2 led to a decrease of ERK signaling, which is a critical element of FGF pathway that inhibits Indian hedgehog homolog (IHH) and parathyroid hormone-related protein (pthrp) expression. Thus, downregulation of ERK significantly increases IHH signaling and promotes malignant proliferation of chondrocytes [49]. Apart from the IHH signaling, inhibition of SHP2 also affects other important chondrogenesis pathways, such as protein kinase A (PKA) and BMP signaling, that is, SHP2 deficiency activates PKA signaling and then stabilizes the SOX9, which is an important transcription factor in chondrogenesis through phosphorylation and SUMOylation. Notably, BMP signaling is also a conserved pathway in the skeleton development, but it is still unclear whether and how SHP2 suppresses BMP pathway [50, 51].

Recently, Qu et al. reported that MSCs with GOF mutation of SHP2 recruit monocytes through CCL3 signaling and monocyte-mediated interlukin-1b pathway to promote HSC malignant proliferation and eventually lead to MPN [14]. Consequently, they further identified that Grb2-associated-binding protein 2 (Gab2), which is a scaffolding protein, is responsible for SHP2 ^E76K^ mutation-caused PI3K/AKT signaling [52]. In summary, SHP2 plays a vital role in MSCs. Yet, the function of SHP2 in MSCs is largely unclear, that is, there are no proofs how SHP2 regulates MSCs trilineage differentiation and/or further interacts with other conserved pathways and is extremely urgent to investigate.

### 1.4. SHP2 in Neural Stem Cells

**Figure 4:**
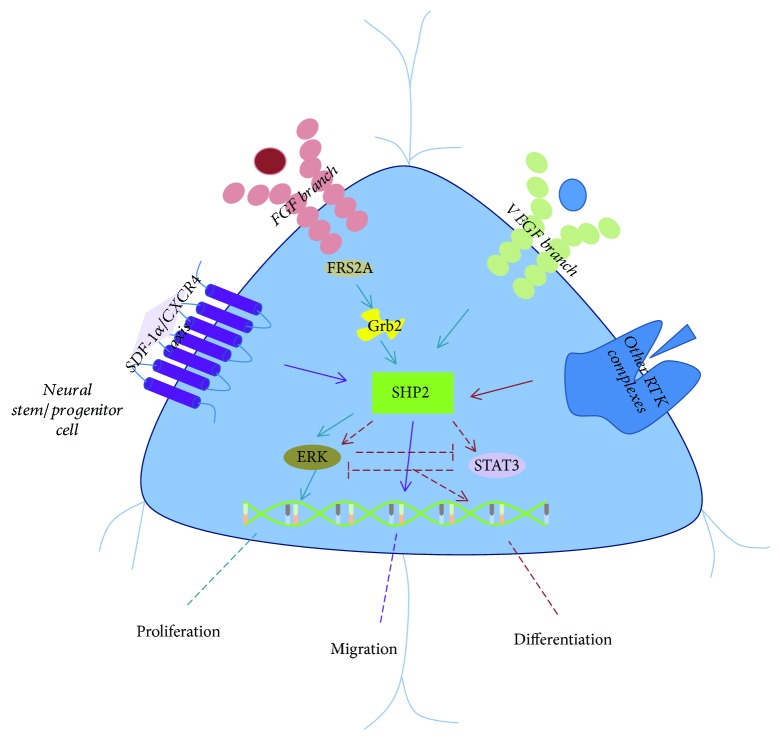
SHP2 plays a key role in NSC proliferation, differentiation, and migration. During NSC proliferation, several essential signaling branches, such as FGF/FGFR and VEGF/VEGFR, transmit the signal to adaptor proteins, for example, Grb2, and subsequently activate SHP2 as well as ERK pathway. Cell cycle-associated genes are considered as the target genes, for example, Bmi-1 is responsible for SHP2-mediated NSC proliferation. Besides, for NSC differentiation, it seems like SHP2 either regulates neurogenesis via ERK signaling or promotes astrogliogenesis through STAT3 signaling. On the other hand, SDF1/CXCR4 is responsible for migration of NSCs and their progenitors.

Neural stem cells (NSCs) primarily differentiate into neurons, astrocytes, and oligodendrocytes and play a central role in brain development and function. Previous studies have indicated that SHP2 was vital to regulate neural stem/progenitor cells proliferation, differentiation, and migration (Figure 4).

SHP2 deficiency reduces oligodendrocyte progenitor cells (OPC) proliferation as well as inhibits oligodendrocytes maturation and myelination [53]. Consistently, ablation of SHP2 in Nestin-cre-labeled cells decreases NSC proliferation and self-renewal. Further in vitro studies showed SHP2-mediated basic FGF signal through control of B lymphoma Mo-MLV insertion region 1 homolog (Bmi-1) which is a regulator of cell cycle [18]. Yamamoto et al. demonstrated that FGF/fibroblast growth factor receptor substrate 2A (FRS2A)/Grb2/SHP2 axis was responsible for NSCs proliferation but not differentiation [54]. Interestingly, Kuo et al. suggested that SHP2 and SHP1 have distinct function in oligodendrocyte development, that is, SHP2 regulates oligodendrocyte progenitor proliferation and SHP1 regulates oligodendrocyte differentiation. ERK1/2 phosphorylation is responsible for SHP2-induced oligodendrocyte progenitor proliferation [55]. For NSCs differentiation, SHP2 was reported to regulate cell fate decisions by promoting neurogenesis while suppressing astrogliogenesis through reciprocal regulation of the ERK and STAT3 signaling pathways [18]. In terms of migration, Huang et al. showed that SHP2-mediated regulation of neural differentiation and migration is related to formation of focal adhesions and activation of RhoA and ERK [56]. Hagihara et al. proposed that SDF-1*α*/CXCR4 signal regulates SHP2 and mediates NSCs-derived cerebellar granule cell migration [57]. Overall, SHP2 also functions as a downstream regulator of several RTKs, such as PDGFR and FGFR, and is closely associated with NSCs homeostasis.

### 1.5. SHP2 in Cancer Stem Cells

**Figure 5:**
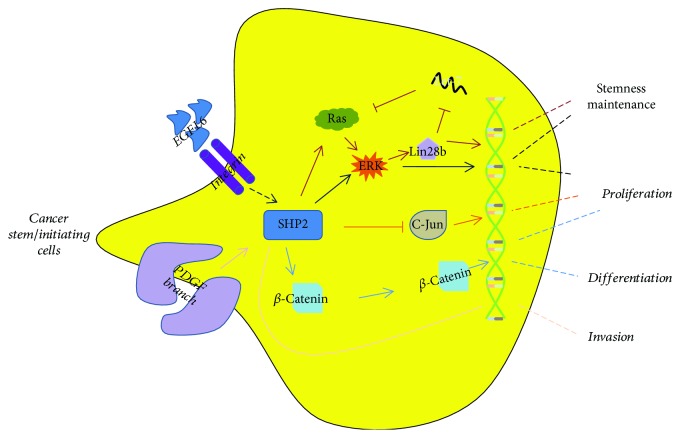
SHP2 differentially regulates CSC function. In different cancer types, SHP2 plays distinct roles. For example, in HER2-positive and triple-negative breast cancers, SHP2 is closely associated with breast cancer initiation and propagation. In addition, SHP2 facilitates glioma stem cell proliferation and invasion. Interestingly, SHP2 could act either as an oncogene or tumor suppressor in primary liver cancer, that is, inhibition of SHP2 promotes C-Jun expression and promotes tumorigenesis, while in chemoresistant hepatocellular carcinomas, increased expression of SHP2 upregulates Wnt signals and facilitates EPCAM^+^ or CD133^+^ cancer stem cell expansion.

Unlike the bulk of adult stem cells, cancer stem cells (CSCs) are thought to initiate tumor growth and cause the recurrence of cancer after chemotherapy and/or radiation therapy [58]. SHP2 is critical to CSCs proliferation and stemness maintenance (Figure 5). For example, simultaneously knockout SHP2 and PTEN in hepatocytes promote liver tumorigenesis. In addition, c-jun, a protooncogene, but not SHP2-associated signal molecules such as ERK and STAT3, is the archcriminal of SHP2/PTEN ablation-induced tumor-initiating cell (TIC) expansion and liver tumorigenesis [59]. Further, SHP2 is also essential in chemoresistant hepatocellular carcinomas. Increased expression of SHP2 upregulates Wnt signals and facilitates EPCAM^+^ or CD133^+^ CSCs expansion by promoting the dedifferentiation of hepatoma cells and enhancing the self-renewal of liver CSCs [17]. The seemingly divergent data about the role of SHP2 may reflect the high heterogeneity of liver cancer. In ovarian cancers, EGFL6, a stem cell regulatory factor expressed in ovarian tumor cells, regulates SHP2 and its concomitant activation of ERK and eventually promotes ALDH^+^ ovarian cancer stem-like cells migration and asymmetric division [60]. In glioma, SHP2 regulates glioma stem cells proliferation and tumorigenicity via targeting SOX2 [61]. Further studies have indicated that PDGFR*α* induces SHP2 upregulation and facilitates glioma cell invasion via ZEB1 signal which is a transcriptional repressor of cell adhesion gene [62]. In human epidermal growth factor receptor 2- (HER2-) positive and triple-negative breast cancers, SHP2 promotes the activation of ERKs including ERK1, ERK2, and ERK5. ERKs induce the oncogenic transcription factors expression such as ZEB1, c-myc, and Lin28b. Lin28b suppresses the expression of let-7 miRNA, which in turn, causes overexpression of let-7 targets, including Ras and C-myc that are closely associated with breast cancer initiation and propagation. This SHP2-dependent positive feedback loop enhances TIC maintenance and breast tumor invasiveness [63]. Overall, SHP2, acted as either an oncogene or tumor suppressor depended on cancer types, is also important for CSCs self-renewal, differentiation, and migration.

## 2. Other Stem Cells

SHP2 is also involved in the regulation of other stem cells, including hair follicle stem cells, satellite cells, intestine stem cells, trophoblast stem cells, and spermatogonial stem cells.

### 2.1. Hair Follicle Stem Cells

Hair follicle contains several stem cells and progenitors including epithelial and melanocyte stem cells. Commonly, we refer the epithelial stem cells located in the bulge region of hair follicles as hair follicle stem cells [64]. Öztürk et al. depicted a complete signal pathway in regulation of hair cycle and hair follicle stem cell quiescence, that is, growth factor-mediated tyrosine kinase receptors (RTK) activated adaptor protein complexes Gab1/Grb2 and subsequently phosphorylated SHP2 and Mek1,2/ERK1,2 cascades and maintained hair follicle stem cells stemness. LOF Gab1 caused exhausted early stem cells and disturbed hair follicle cell cycle [65].

### 2.2. Satellite Cells

Satellite cells, also called muscle stem/progenitor cells, are small multipotent cells with virtually no cytoplasm found in mature muscle. SHP2 also regulates satellite cells proliferation and differentiation, that is, LOF SHP2 drives satellite cells into quiescence and upregulation of SHP2 activates ERK signal and is responsible for satellite cells proliferation. In addition, SHP2 enhances MyoD/MyoG signaling which is essential in the coordination of skeletal muscle development or myogenesis and repair and directs satellite cells differentiation into muscle cells [66].

### 2.3. Intestinal Stem Cells

In the adult intestine, epithelial stem/progenitor cells located in the niche of the crypts of Lieberkühn are intestinal stem cells. Heuberger et al. found that in the leucine-rich repeat-containing G-protein-coupled receptor 5 (Lgr5)^+^ intestinal stem cells, upregulation of SHP2 activates dual specificity mitogen-activated protein kinase kinase 1 (Mek1), which in turn phosphorylates ERK and promotes goblet cell differentiation. In contrast, inhibition of SHP2 causes ERK suppression and increases the Wnt/*β*-catenin signaling; hyperactivation of Wnt signal promotes Paneth cell differentiation and the maintenance of stem cells stemness [67].

### 2.4. Trophoblast Stem Cells

Trophoblast stem cells are the stem/progenitor cells for differentiated trophoblast derivatives and reside above the inner cell mass which is located on one side of a cystic cavity. In trophoblast stem cells, SHP2 is also an important mediator of RTKs, for example, FGF receptor. FGF4-evoked RTK signals activate the protooncogene protein tyrosine kinase Src and then Ras and ERK pathway. This effect promotes Bcl-2-like protein 11-mediated proapoptotic function and eventually regulates stem cells proliferation and apoptosis [68].

### 2.5. Spermatogonial Stem Cells

Spermatogonial stem cells (SSCs) are the early precursor for spermatozoa and are responsible for the continuation of spermatogenesis in adult mammal. Ablation of SHP2 leads to excessive differentiation of SSCs by disturbing the expression of paracrine factors and ERK signaling. Therefore, SHP2 is essential to maintain Sertoli cell function [69].

## 3. Conclusion

Overall, SHP2 seems to play a key role in stem cells homeostasis. Although many conserved signal pathways have been reported to regulate SHP2 function, not all pathways contribute equally to stem cells self-renewal and differentiation (Table 1). The dominant mechanisms are likely context dependent. For example, SHP2-ERK signaling mediates ESCs endoderm lineage differentiation, but in HSCs, it is responsible for proliferation. In addition, SDF1/CXCR4 activates SHP2 and promotes NSCs migration. Despite this complexity, some conclusions can be drawn: (1) SHP2 is a mediator between external stimulation and nuclear responses, and several RTKs are critical as a trigger in this case. (2) Scaffolding adaptor proteins, such as Gabs and Grbs, are also responsible for SHP2 activation, and consequently, SHP2 impacts downstream cascades like ERK, C-jun, GATA, and STAT and eventually regulates various targeted gene expression. (3) Many signal cascades regulate stem cells capacities through cross-talking with SHP2. (4) SHP2 is considered as a protooncogene in leukemia and several cancer types. (5) SHP2 is also considered as a target gene in regenerative medicine. (6) SHP2 might also have direct implications for MSC-associated diseases, such as HO.

## Figures and Tables

**Table 1:** Similarities and differences between SHP2 signaling in different stem cells.

	SHP2-ERK signaling	SHP2-STAT signaling	SHP2-P38 signaling	SHP2-C-jun signaling	SHP2-AKT signaling
ESCs	Ectoderm and/or endoderm lineage differentiation	Self-renewal	Mesoderm lineage differentiation	Not determined	Not determined
HSCs	Self-renewal	Proliferation	Not determined	Monocytic differentiation	Proliferation
MSCs	Chondrogenic differentiation	Not determined	Not determined	Not determined	Proliferation and osteogenic differentiation
NSCs	Proliferation and/or neuron differentiation	Astrocyte differentiation	Not determined	Not determined	Not determined
CSCs	Self-renewal and differentiation	Not determined	Not determined	Proliferation	Not determined
